# Detection of Defects in Geomembranes Using Quasi-Active Infrared Thermography

**DOI:** 10.3390/s21165365

**Published:** 2021-08-09

**Authors:** Yue Ma, Francis Rose, Leslie Wong, Benjamin Steven Vien, Thomas Kuen, Nik Rajic, Jayantha Kodikara, Wingkong Chiu

**Affiliations:** 1 Department of Mechanical and Aerospace Engineering, Monash University, Wellington Rd, Clayton, VIC 3800, Australia; leslie.wong@monash.edu (L.W.); ben.vien@monash.edu (B.S.V.); Wing.Kong.Chiu@monash.edu (W.C.); 2 Defence Science and Technology Group, 506 Lorimer St., Fishermans Bend, VIC 3207, Australia; Francis.Rose@dst.defence.gov.au (F.R.); Nik.Rajic@dst.defence.gov.au (N.R.); 3 Melbourne Water Corporation, 990 La Trobe Street, Docklands, VIC 3008, Australia; Thomas.Kuen@melbournewater.com.au; 4 Department of Civil Engineering, Monash University, Clayton, VIC 3800, Australia; Jayantha.Kodikara@monash.edu

**Keywords:** non-contact inspection, quasi-active thermography, HDPE geomembrane, defect detection, floating covers, thermal image processing

## Abstract

High-density polyethylene geomembranes are employed as covers for the sewage treatment lagoons at Melbourne Water Corporation’s Western Treatment Plant, to harvest the biogas produced during anaerobic degradation, which is then used to generate electricity. Due to its size, inspecting the cover for defects, particularly subsurface defects, can be challenging, as well as the potential for the underside of the membrane to come into contact with different substrates, viz. liquid sewage, scum (consolidated solid matter), and biogas. This paper presents the application of a novel quasi-active thermography inspection method for subsurface defect detection in the geomembrane. The proposed approach utilises ambient sunlight as the input thermal energy and cloud shading as the trigger for thermal transients. Outdoor laboratory-scale experiments were conducted to study the proposed inspection technique. A pyranometer was used to measure the intensity of solar radiation, and an infrared thermal camera was used to measure the surface temperature of the geomembrane. The measured temperature profile was analysed using three different algorithms for thermal transient analysis, based on (i) the cooling constant from Newton’s law of cooling, (ii) the peak value of the logarithmic second derivative, and (iii) a frame subtraction method. The outcomes from each algorithm were examined and compared. The results show that, while each algorithm has some limitations, when used in combination the three algorithms could be used to distinguish between different substrates and to determine the presence of subsurface defects.

## 1. Introduction

**Figure 1:**
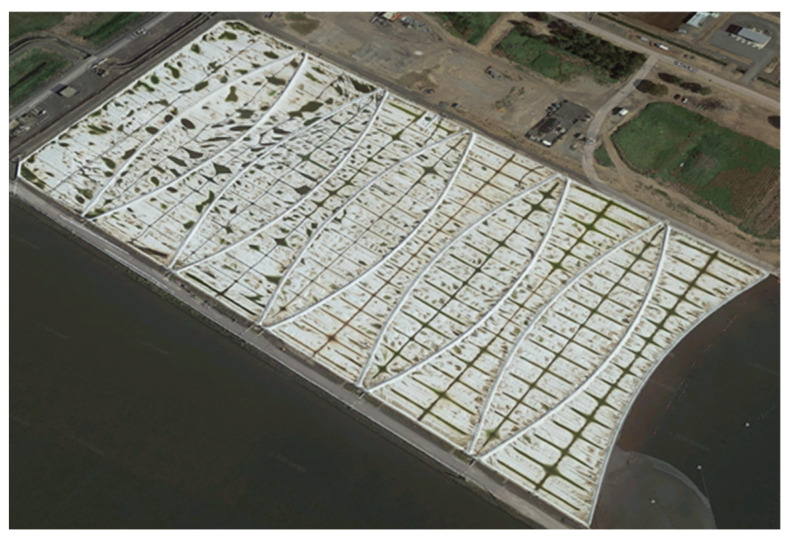
Illustration of HDPE floating cover in the WTP.

A high-density polyethylene (HDPE) geomembrane (called the “membrane” in this paper) has the advantages of high stiffness and tensile strength, as well as good chemical resistance. Such membranes have been extensively applied in sewage treatment plants, landfill covers, and waste degradation plants as a cover material to prevent the diffusion of contaminants [1]. In the Western Treatment Plant (WTP) of the Melbourne Water Corporation (MWC), Victoria, Australia, several large-scale HDPE geomembranes are deployed as floating covers on anaerobic degradation lagoons. As shown in Figure 1, these 2 mm thick membranes, spanning an area of approximately 475 m × 216 m, are installed on the top of lagoons where they float on raw sewage. These covers capture the anaerobic processes that go on in these lagoons and release biogas, odours, and greenhouses gases. These biogases also constitute a renewable energy source and each floating cover can capture up to 65,000 m^3^ of biogas per day, which is used to produce 7 MW of electrical energy [2].

The expected service life of the membrane is from 50–90 years at an operating temperature of 50 °C, and more than a thousand years at 20 °C [3]. However, due to the high crystallinity of the membrane, its impact strength and stress cracking strength are compromised by the operational environment. Stress cracks usually occur early in the life of membranes in high-stress conditions [4]. At the WTP, unfiltered raw sewage flows into the covered end of the lagoons. During the anaerobic digestion of the sewage, a combination of floatable solids, fats, oils, greases (FOG), fibrous materials, and low-density sludge creates a “scum” that can entrap small bubbles of biogas and then be transported up through the sewage to the underside of the cover. The accumulation of scum can increase to various volumes and hardness. Some may stick to the underside of the cover and/or solidify over time [2]. Large accumulations of consolidated solid matter under the cover are called scumbergs. Scumbergs can adversely affect the structural integrity of a floating cover in the following ways:

(1) The motion of hard scumbergs underneath the membrane, typically caused by the inflow of raw sewage into the lagoons, can produce defects on the underside that are not readily detected by visual inspection; these will be referred to as non-surface-penetrating defects;
(2) Large scumbergs can lift the membrane above the surface level of the sewage. Combined with the wind blowing over the elevated cover, this can laterally stretch the membrane, thus producing large strains and increasing the damaging potential of non-surface-penetrating defects.

The challenge is to be able to detect and quantify the structural significance of these non-surface-penetrating defects. Currently, the only way to identify defects in the cover at the WTP is by walking over it. This technique cannot detect underside surface defects. A non-contact inspection methodology is preferred because of the generation of biogas under the cover. As biogas is flammable, there is a very strong preference that any equipment taken out and used above the cover be intrinsically safe, or that any work that has the potential to generate heat or an ignition source is performed under a “Hot Work Permit” system which includes a thorough Task Risk Assessment and review and approval process. The non-gas substrates (i.e., liquid and solid-state matter) increase the difficulties in detecting the presence of membrane defects. There are limits in applying a stimulus and monitoring the vibrational response using contact inspection techniques due to the large size of a membrane (more than 8 hectares [5]). This paper presents a novel detection strategy based on a non-contact thermal method that utilizes the phenomenon of naturally occurring variations in solar intensity to enable the inspection of floating covers. This inspection technique has the potential to be an integral part of a structural health monitoring regime for this large asset, providing early warning of damage, thereby enabling more efficient asset management and maintenance of the cover.

In existing thermography monitoring studies, the thermographic inspection of large structures is mostly undertaken using ambient sunlight as the stimulus for interrogation, and the inspection result mainly depends on the temperature contrast within a single frame of the thermal image. Omar and Nehdi [6] mounted an infrared (IR) camera on an unmanned aerial vehicle (UAV) to remotely monitor the temperature distribution on bridges when heated by sunlight. Thermal images of each segment of the bridge were stitched together and used to generate a temperature map of the entire bridge. They used the K-mean clustering machine learning method to enhance the contrast of these thermal images. They also developed a vehicle-mounted IR camera system to monitor the delamination in bridge structures using sunlight as the stimulus [7]. A thermal camera mounted on the back of a vehicle was used to scan the temperature distribution along the path traversed by the vehicle. The delamination was revealed by the presence of abnormal temperature gradients stimulated by the radiant heating from the sun. Teza [8] deployed fixed IR cameras to monitor the cracked part of the walls of two historical towers, where the defective parts were identified through thermal contrast.

This paper presents a fresh approach for detecting non-surface-penetrating defects, based on quantifying the cooling kinetics during transients caused by variations in solar intensity. This approach is compared with two conventional thermographic approaches, viz. peak logarithmic second derivative and frame subtraction [9,10,11]. The theoretical underpinning of these approaches is summarised in Section 3. The focus for the present paper is an experimental evaluation of these approaches for synthetic defects in a membrane that is in contact with either water (simulating liquid sewage), or garden soil (simulating scum), or air (simulating pockets of biogas). The experiments were conducted in an open space using sunlight intensity fluctuations from short periods of cloud shading, as described in Section 2. The relative merits and limitations of these approaches are briefly discussed in Section 4.

## 2. IR Thermography Inspection of HDPE Geomembranes

### 2.1. Quasi-Active Thermography

Thermographic inspection techniques can be generally classified as either active thermography or passive thermography. Active thermography [12,13,14,15] uses an external heat stimulus, such as a halogen lamp or a hot air gun, to inject thermal energy into the object under inspection. The technique relies on heat flow in the inspected object being interrupted by a defect thereby leading to a thermal contrast relative to an undamaged region. Passive thermography [16,17,18] uses naturally occurring temperature fluctuations or cooling processes as a basis for monitoring. The technique relies on the temperature of monitored objects being different from the surroundings, resulting in heat exchange between the object and its surroundings. Defects, or anomalies, within parts subject to such heat exchange can often generate a detectable thermal contrast. One of the more common practical applications of passive thermography is for the inspection of entrapped water in honeycomb-reinforced structural components [19]. In this case, inspectors examine an aircraft immediately after landing, on the expectation that any entrapped water would have frozen at altitude and would show up as a cold spot.

The thermographic inspection method investigated in the present work uses solar radiation or sunlight as the interrogating heat source and specifically takes advantage of periods of disrupted irradiance due to cloud cover to induce thermal transients in the membrane. V.P. Vavilov et al. used thermal imaging to detect the buried landmines [20]. A complex character of solar irradiation and its influence on detecting landmines in soil were discussed, and the relation between the surface temperature and various parameters were investigated.

A transient event occurs when cloud shading reduces sunlight intensity on the membrane. The temperature of the membrane decreases due to a decline of input heat. If a defect alters the heat-transfer characteristics of the membrane, this will result in a local variation in the temperature distribution on the membrane surface, and hence, on its IR emission. During the cloud shading, the temperature of the geomembrane reduces due to the decreased power of radiation, but the external heat is continuously applied on the surface of the geomembrane. The “cooling” in this paper refers to the reduction in the geomembrane temperature during the cloud shading event. When the sun reappears from the cloud another transient event occurs in response to an increase in the local solar intensity. These temperature transients are similar to those induced in pulse thermography [12,21], but using a naturally occurring stimulus. Accordingly, our approach can be aptly described as quasi-active thermography.

**Figure 2:**
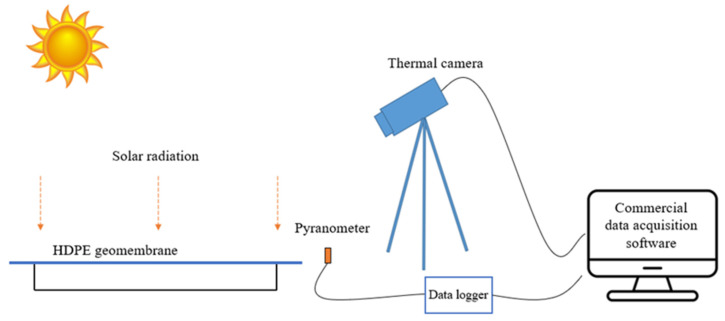
Illustration of data acquisition phase in the quasi-active thermography experiment.

The transient events caused by the cloud shadings can happen many times in a day, with the temperature gradients in the membrane depending on the duration of cloud shading and the density of the clouds. In our experiments, the local solar radiation intensity and the temperature of the membrane are recorded by a pyranometer and a thermal camera, respectively, as shown in Figure 2. The measured temperature-decay curves at each location are employed to derive a cooling constant as discussed below in Section 3, and a resulting map of this cooling constant provides the basis for finding defects or the boundaries between different substrates.

### 2.2. Emissivity Assessment of HDPE Geomembrane

Objects such as membranes are observed to radiate heat in accordance with the Stefan–Boltzmann law, modified for grey-body radiation [22,23], viz:

(1) $$Q_{rad}=\epsilon \sigma T^{4}$$

where $Q_{rad}$ is the power flux per unit area from the surface, ϵ is the emissivity of the surface of the object, *σ* is the Stefan–Boltzmann constant, and *T* is the absolute temperature of the surface. To determine the suitability of using IR thermography to monitor the membrane, the emissivity spectrum of the membrane specimens from the WTP covers has been measured using Fourier Transform Infrared (FTIR) spectroscopy in previous research [24]. The emissivity of the membrane was found to range between 0.94 and 0.95 in the long-wavelength IR spectrum, which indicates that the membrane is a strong emitter of thermal radiation, and, therefore, suitable for inspection by IR thermography.

### 2.3. Experimental Set-Up

Figure 2 illustrates the integrated data acquisition set-up for quasi-active thermography. Two membranes were sourced from the WTP (same as the actual membrane) and fixed in turn on a 110 cm (L) × 110 cm (W) × 10 cm (D) aluminium test rig with screws. An IR thermal camera FLIR A615 [25] and a pyranometer Apogee SP-110 [26] were set up 0.5 m horizontally away from the test rig to measure the surface temperature profile of the membrane and the local solar intensity, respectively. To validate the infrared thermal imaging accuracy, the measured temperature from the thermal camera was at first compared with a FLUKE 287 multimeter contacting thermal probe. A commercial software package was employed to control the thermal camera for recording the temperature evolution of the membrane. The Apogee SP-110 pyranometer was placed on the same level beside the thermal camera (set up 1 m above floor level). The technical specifications for the FLIR A615 camera, SP-110 pyranometer, and Fluke 287 multimeter thermal probe are provided in Table 1, Table 2 and Table 3, respectively. The pyranometer was connected to a data logger and the solar intensity was acquired in real-time and time-stamped using commercial data acquisition software. To ensure a synchronous profile for the data, the sampling rate of the pyranometer was set to be the same as the frame rate of the thermal camera (3 Hz). The outdoor laboratory-scaled experiment was set up on the rooftop of a building at Monash University, where the test set-up is not susceptible to any shading from nearby structures or buildings.

### 2.4. Defects in the HDPE Geomembrane with Various Substrates

The aim of this study is to assess the ability of quasi-active thermography to detect underside non-surface-penetrating defects lying over various substrates. In the present experimental work, garden soil was used to simulate scum because its relevant properties (i.e., density, thermal conductivity, and specific heat) are similar to those of solid scum, as summarised in Table 4, where the thermal diffusivities of each material can be calculated through the given density, thermal conductivity, and specific heat [27,28,29,30,31,32]. Therefore, the membrane in this experiment was placed in contact with soil, water, and air to mimic, respectively, contact with scum, sewage, and biogas in the treatment plant.

**Figure 3:**
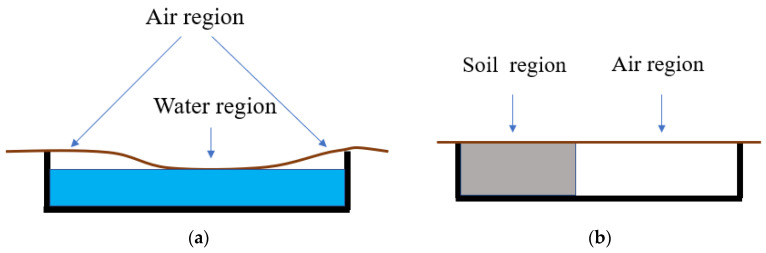
Side profiles of test rig. (**a**) membrane on air and water; (**b**) membrane on air and soil.

Figure 3a,b show schematic drawings of the cross-sectional view of the two membranes and the test rig with different substrate configurations. In Figure 3a, a part of the membrane was brought into contact with water with the rest suspended in air. This simulates biogas pockets under the WTP floating covers. In Figure 3b, one part of the membrane is in contact with a soil block, which simulates a region of scum, and the other part is suspended in air.

**Figure 4:**
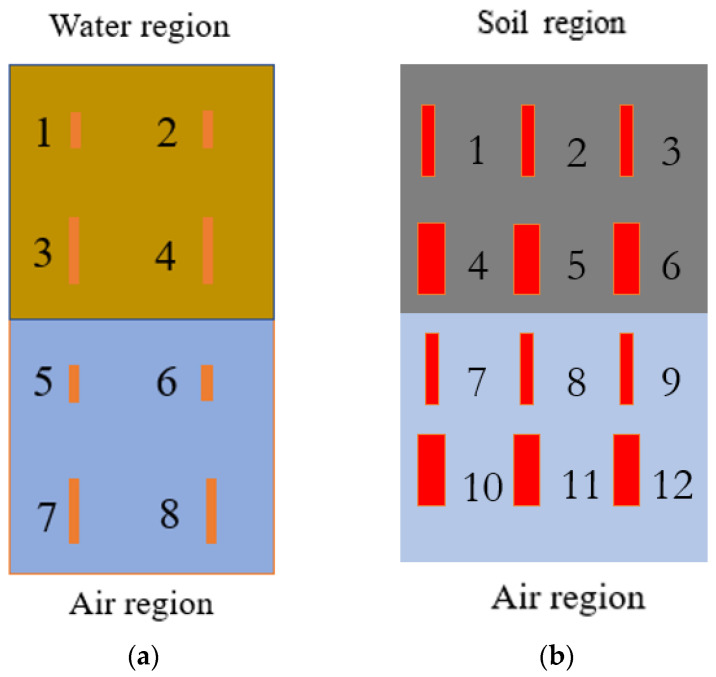
Distribution of defects in the membrane (plan view): (**a**) defects in contact with water and air; (**b**) defects in contact with soil and air.

**Figure 5:**
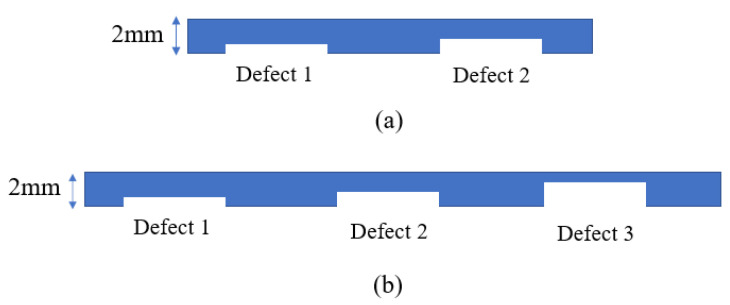
Cross-sectional view of two membranes: (**a**) profiles of defect 1 and defect 2 in Figure 4a; (**b**) profiles of defect 1, 2, and 3 in Figure 4b.

Defects of different sizes were manufactured into the underside surface of two separate membranes by laser cutting. In the floating covers at the WTP, there is a potential that the underside of the geomembrane can be damaged by the movement of scums. The laser cutting defects in this paper are used to simulate the occurrence of this type of defect. Figure 4a,b show a plan view (top) of the distribution of synthetic defects on the underside of two different membranes, where the geometry of each defect is detailed in Table 5 and Table 6, respectively. As shown in Figure 4a, on the first membrane, defects 1–4 were in contact with water, and defects 5–8 were in contact with air. In Figure 4b, defects 1–6 were in contact with soil, and defects 7–12 were in contact with air. Figure 5 further illustrates the profiles of defects with a cross-sectional view. The two membranes were fixed on the aluminium test rig and left exposed to solar radiation during the daytime for several hours, while transient events resulting from cloud shading were recorded using the IR thermal camera and pyranometer set-up described in Section 2.3.

## 3. Thermographic Data Analyses

Data analysis for pulsed thermography generally relies on tracking the surface temperature difference between a potentially defective location and a reference location that is presumed to be defect-free [9,10,11]. This temperature contrast curve typically shows a maximum at a particular time instant, known as the peak contrast time (PCT), that is approximately proportional to the square of the defect depth beneath the surface. The first derivative of the thermal contrast also shows a peak, at a time instant known as the peak slope time (PST), that also correlates approximately with the square of defect depth. In addition to these approaches, Shepard et al. [9] proposed a reference-free approach that relies solely on the temperature evolution at a given point. Their approach evaluates the second derivative of temperature in the logarithmic domain, employing a logarithmic scale for both temperature and time. The peak time for this logarithmic second derivative also correlates approximately with defect depth, and because this peak occurs earlier in time than PCT and PST, the effects of blurring (due to lateral heat diffusion) are reduced, thereby resulting in clearer delineations of defective regions. Another processing method commonly applied is principal component thermography [37], which uses a singular value decomposition of the thermal response to accentuate defect thermal contrast.

This paper proposes an alternative approach based on tracking the cooling kinetics during a decrease in solar intensity due to cloud cover, following a period of sustained heating. Although the cloud shading events are not completely step-wise, the transient responses during these events are used to identify defects on the floating membrane. For a membrane on air (or biogas), the cooling kinetics is controlled by Newton’s law of cooling, as summarised below in Section 3.1. While this cooling law does not strictly apply for a membrane in contact with water or soil (simulating liquid sewage or scum, respectively), it is still possible to extract a cooling constant by fitting the data to an assumed exponential decay curve. The expectation is that subsurface defects may lead to detectable spatial variations in this cooling constant. For completeness, the data processing algorithms for the peak logarithmic second derivative and frame subtraction are also briefly recalled below.

### 3.1. Newton’s Cooling Law Method

Heat transfer from a hot membrane in contact with air occurs by convection, $q_{conv}$ and radiation,$\;q_{rad}$, i.e.:

(2) $$q=q_{conv}+q_{rad}$$

where *q* denotes the combined heat flux, and the subscripts identify the two contributions. According to Newton’s law of cooling [22,23],

(3) $$q_{conv}=h_{c}\left[T\left(t\right)-T_{a}\left(t\right)\right]\;$$

where $T,T_{a}$ denote, respectively, the membrane temperature and ambient temperature at a time instant *t*, and $h_{c}$ denotes the convective heat transfer coefficient.

Radiative heat transfer satisfies Equation (1). Allowing for a radiative heat flux from the surroundings, the net radiative heat flux at the surface of a membrane can be written as follows [22,23]:

(4) $$q_{rad}=h_{r}\varepsilon \sigma \left(T^{4}-T_{a}^{4}\right)$$

where $T,T_{a}$ must now be interpreted as absolute temperatures (in accordance with Equation (1)), and Kirchoff’s law, equating absorptivity α with emissivity ε has been invoked. For the range of temperatures encountered in the present context, the difference $T-T_{a}$ is relatively small compared with the absolute ambient temperature $T_{a}$, so that Equation (4) can be rewritten in the form:

(5a) $$q_{rad}=h_{r}\left[T\left(t\right)-T_{a}\left(t\right)\right]$$

(5b) $$h_{r}\approx 4\varepsilon \sigma T_{a}^{3}\;$$

Accordingly, Equation (2) can now be rewritten as:

(6a) $$q=h\left[T\left(t\right)-T_{a}\left(t\right)\right]$$

(6b) $$h=h_{c}+h_{r}$$

The next step in characterising the cooling kinetics is to estimate the Biot number:

(7) $$Bi=\frac{hL_{c}}{k}$$

where *k* denotes the thermal conductivity and $L_{c}$ denotes an appropriate characteristic length, which in the present case, can be assumed to be the membrane thickness (rather than the more common use of the half-thickness, consistent with the assumption of zero heat flux from the underside of the membrane). Employing $h=13\;{\mathrm{Wm}}^{-2}K^{-1}$ as a representative value for the total heat transfer coefficient for a horizontal membrane [36], $k=0.44\;{\mathrm{Wm}}^{-1}K^{-1}$ for the conductivity of HDPE [33], and $L_{c}=2\;\mathrm{mm}$ leads to Bi≈0.06. As this value is less than 0.1, the temperature gradient across the membrane thickness can be ignored [22,23]. Accordingly, the heat balance for a membrane on air (or biogas) can be written as follows:

(8) $$\rho CL\frac{dT\left(t\right)}{dt}=h\left[T\left(t\right)-T_{a}\left(t\right)\right]$$

where ρ,C,L denote, respectively, the membrane’s density, specific heat, and thickness. Assuming, for simplicity, that the ambient temperature $T_{a}$ can be regarded as being constant, or only slowly time-varying, Equation (8) can be integrated to obtain:

(9a) $$T\left(t\right)=T_{a}+\left(T_{i}-T_{a}\right)e^{-bt}$$

(9b) $$\tau =b^{-1}=\frac{\rho CL}{h}$$

where $T_{i}$ denotes the initial temperature, and *b* the cooling constant. Using the parameter values given above (cf. Table 4) leads to the following estimate for the time constant (the reciprocal of the cooling constant *b*), τ≈270 s.

Although Equation (9) has been derived by assuming that the ambient temperature remains constant, τ nevertheless provides a useful characteristic time, e.g., for setting a time interval for assessing the thermal contrast, or for discarding transient events that occur on a time scale much shorter than τ.

For a membrane in thermal contact with a substrate such as water or soil, there is a significant heat transfer by conduction to the substrate (as there would also be for liquid sewage or scum, cf. Table 4). Accordingly, the above estimate for the Biot number is not applicable, and Equations (8) and (9) are also not strictly applicable. Nevertheless, temperature decay profiles $T_{\left(i,j\right)}$ recorded for each pixel (*i*,*j*) in the region of interest can be curve-fitted to Newton’s cooling law, Equation (9), to obtain a corresponding cooling constant, $b_{\left(i,j\right)}$ for each pixel location, in the expectation that spatial variations in this fitted constant may serve to identify regions with subsurface defects.

### 3.2. Peak Logarithmic Second Derivative Method

The logarithmic second-order time derivative of the temperature $T\left(x,y,t\right)$ is given by [9]:

(10) $$J\left(x,y,t\right)=\frac{d^{2}\left(\ln T\right)}{d{\left(\ln t\right)}^{2}}=\frac{t}{T}\frac{dT}{dt}-\frac{t^{2}}{T^{2}}\frac{d^{2}T}{dt^{2}}+\frac{t^{2}}{T}{\left(\frac{dT}{dt}\right)}^{2}$$

For the case of pulsed thermography, Shepard et al. [9] have shown that *J* reaches a maximum value $J_{max}$ at a characteristic time that correlates with the square of defect depth, but occurs earlier than PCT or PST, thereby reducing the extent of blurring due to lateral heat diffusion, which leads to a clearer delineation of defective regions. In their implementation, the experimental temperature data is fitted by a low order polynomial in the logarithmic domain, which serves as a low-pass filter that preserves the essential thermal response, while also providing an analytical representation for *J*.

In outdoors thermal imaging, the ambient temperature is not constant. In addition, the external heat flux (solar radiation) is expected to fluctuate during the cloud covers events. Therefore, the characteristic time cannot be used to correlate the thickness of the depth in the outdoors thermal imaging. In the present work, the peak logarithmic second derivative method was modified by using the values of $J_{max}$ to estimate the profiles of substrates and the defects. The Savitzky–Golay algorithm is employed, with a window size of 11, for data smoothing and for calculating the derivatives on the right-hand side of Equation (10). At a 3 Hz sampling rate, this represents an approximate 4 s window, which is considered to be short enough to avoid biasing the derivative calculation. The resulting value of $J_{max}$ is plotted and the resulting map is assessed as a basis for identifying subsurface defects.

### 3.3. Frame Subtraction Method

In this method, an image is obtained by subtracting the temperature field at a time instant $t_{n}$ from that at an earlier time $t_{n-1}$ in the cooling sequence, and the absolute values of temperature changes over the whole event are summed up to produce an image *I*(*x*,*y*), i.e.:

(11a) $$I\left(x,y\right)=\overset{N}{\underset{n=1}{\sum }}\left|T\left(x,y,t_{n-1}\right)-T\left(x,y,t_{n}\right)\right|$$

where

(11b) $$t_{n}=t_{0}+n\times \Delta t$$

and $t_{0}$ denotes the chosen start point for the transient event, whereas $t_{N}$ denotes the chosen end point of the transient event.

It is noted that if the temperature *T* is a strictly monotonically decreasing function of time, Equation (11) reduces to:

(12) $$I\left(x,y\right)=T\left(x,y,t_{0}\right)-T\left(x,y,t_{N}\right)$$

i.e., the resulting image would only depend on the start and end points, and not on the choice of the time interval Δt. However, in the present context, there are short time-scale fluctuations in solar radiation that can result in short time-scale increases in temperature during the course of what is, overall, a cooling transient. These short time-scale fluctuations are captured when using a time interval Δt=10 s, and it was found that by using the absolute value of the temperature difference between successive time instants, as indicated in Equation (11), one obtains an enhanced contrast between defective versus intact regions, relative to what would be obtained from Equation (12), as shown below in Section 4.

## 4. Results and Discussion

Two experimental cases were considered as previously described, viz.:

- Case 1: a membrane with water and air substrate (Figure 3a);
- Case 2: a membrane with soil and air substrate (Figure 3b).

For both cases, thermal image sequences were acquired at a frame rate of 3 Hz, but to reduce the computational burden of analysis, sequences were down-sampled to an effective rate of 0.1 Hz prior to processing.

### 4.1. Case 1: Inspection of Defects on Water and Air Substrates

**Figure 6:**
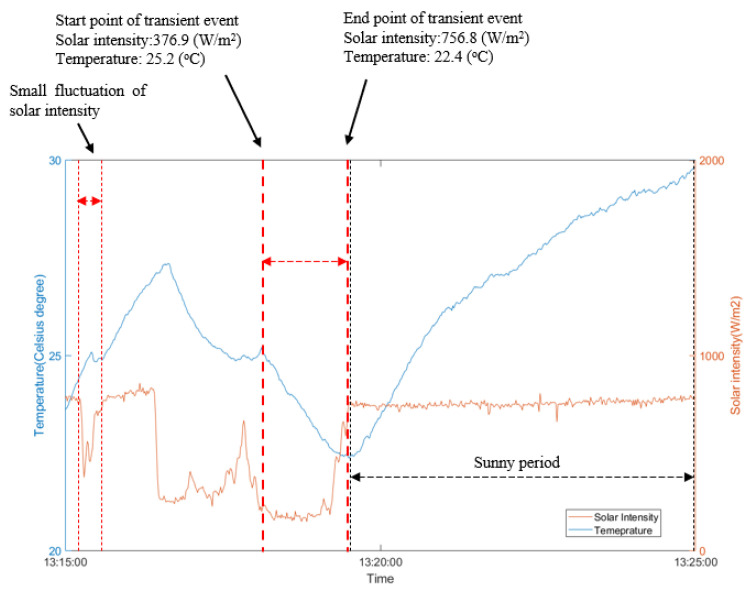
Temperature profile from a pixel on no-water region in the thermal image sequences and solar intensity history.

The first inspection for Case 1 comprised an observation lasting 20 min, starting at 13:15:00, as shown in Figure 6. Three cloud shadings events were observed within this time. Not all the cloud shading events were taken into consideration because some did not cause a significant temperature transient. For example, Figure 6 shows a small solar intensity fluctuation within a short time (less than 10 s) 1 min after the start of the experiment, inducing only a subtle change in temperature (~0.1 °C) in the membrane. The solar radiation during cloud cover events comprises a series of local maxima (i.e., local heating during the overall cooling event). Such events were therefore excluded from the analysis. In the thermography of a 2 mm thick HDPE membrane, a more significant temperature reduction is required for detection. Therefore, a pragmatic criterion for selecting a useful event for analysis is that the solar intensity should be reduced by more than 400 W/m^2^ and cloud shading should last more than 1 min.

Figure 6 shows a desirable cloud shading event starting at approximately 13:18:08 and ending at 13:19:30 (a duration of 82 s), during which the local solar intensity reduced, resulting in the temperature of the membrane falling from 25.2 °C to 22.4 °C. The temperature decay curves for all pixels from the highlighted event between the dashed lines in Figure 6 were analysed using the three analysis methods described in Section 3.

**Figure 7:**
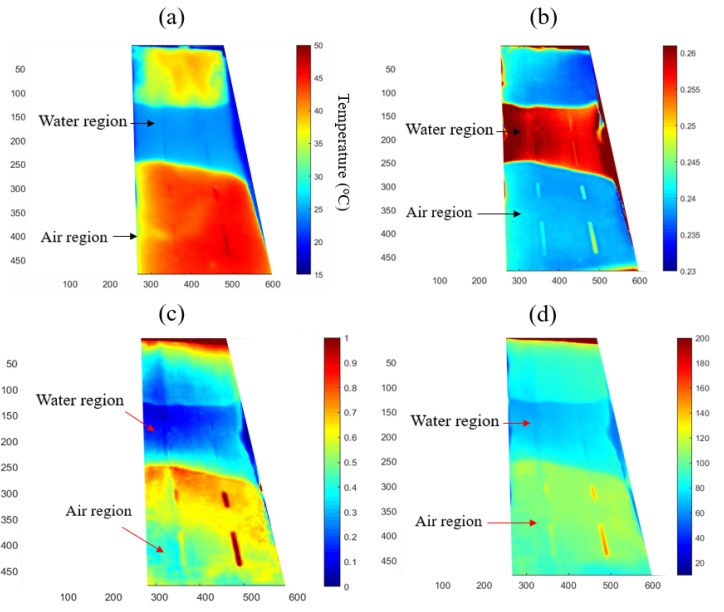
Results of analysis of thermal image sequences with different methods: (**a**) a single frame of the raw thermal image, (**b**) cooling constant map based on Newton’s cooling law analysis, (**c**) peak logarithmic second derivative method, and (**d**) frame subtraction method.

The results are shown in Figure 7. They are evaluated based on: (1) the ability to distinguish between the substrates; and (2) the ability to identify subsurface defects. The raw thermal image, shown in Figure 7a was taken at the midpoint of the transient event. In this image, the water region can be distinguished due to its significantly lower temperature, the defects are visible but their profiles are not clear.

Figure 7b shows a map of the cooling constant obtained by the approach described in Section 3.1, based on a calculation encompassing the whole cooling period. The membrane and water regions are more distinct in this result, and the defects in the region with an air substrate produce easily discernible signatures. Defects number 6 and 8 (as identified in Figure 4a) exhibit more contrast than defects 5 and 7, which correlates with them being nearer the surface. In the membrane-water region, defects 2 and 4 (cf. Figure 4b) produce a somewhat weaker contrast that is barely discernible, whereas Defects 1 and 3 are barely visible but visible nonetheless, the former less so because it is partially obscured by a vertical strip artefact, which is consistent with the small size of those defects. Thus, it can be concluded that Newton’s cooling law method provides a more effective approach for damage detection compared with the visual inspection of a single image frame.

Figure 7c illustrates a map of the peak value of the logarithmic second derivative, as presented in Section 3.2. It successfully shows the boundaries between the water and air regions, and there is sufficient contrast to detect defects 5 to 8 in the air region, whereas defects in the water region are barely visible. The result from the frame subtraction method is plotted in Figure 7d. Similar to Figure 7c, the boundary between the water region and the air region can be identified via the contrast. Defects numbers from 5 to 8 in the air region can be identified in Figure 7d, and there is also a faint indication of defects 2 and 4 in the water region.

This comparison of the merits and limitations of the three methods is summarised in Table 7. Although Figure 7 can present profiles of defects on different substrates, it is recommended in future studies to use a fuzzy image pre-processing technique [38,39] to better delineate the contours between the individual areas.

### 4.2. Case 2: Inspection of Defects on Soil and Air Substrates

For the second case study, the water was drained from the previous set up and garden soil was compacted in the aluminium test rig before being covered by the membrane, ensuring good thermal contact between the membrane and soil. The test set-up was left outdoors overnight to ensure thermal equilibrium.

**Figure 8:**
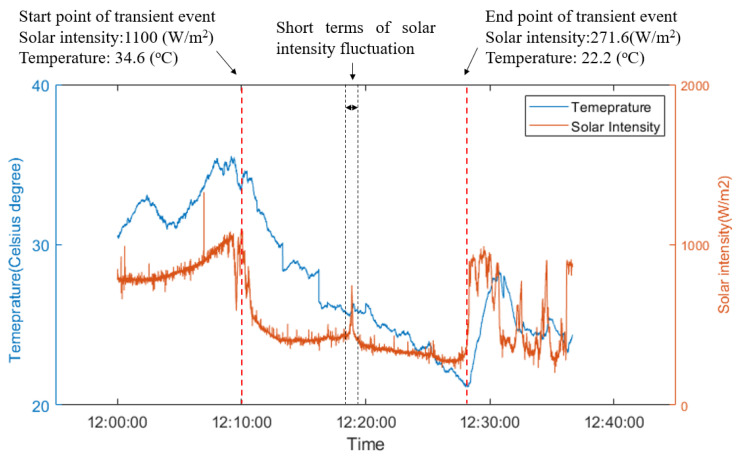
Temperature profile from a pixel on the air region in thermal image sequences and solar intensity history.

The quasi-active thermal inspection commenced at 12:00:00 during a period of high solar intensity, as shown in Figure 8. A cloud shading event occurred from 12:10:00 to 12:27:45. Local solar intensity decreased from 1100 W/m^2^ to 271.6 W/m^2^ (a change of 828 W/m^2^). This event resulted in a temperature reduction of 12.4 °C, which was enough for the thermal transient analysis. A short fluctuation in solar intensity was also noted at 12:18:00 during the transient event, but it lasted less than 30 s and did not lead to a significant change in the temperature response of the membrane. Therefore, this fluctuation can be ignored for the purposes of transient thermal analysis.

**Figure 9:**
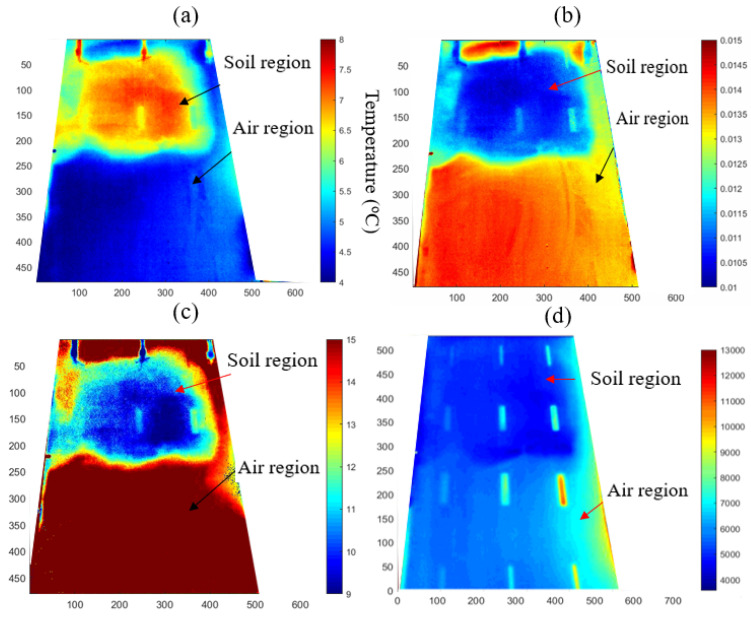
Results of analysis of thermal image sequences. (**a**) single frame of the raw thermal image, (**b**) cooling constant map of Newton’s cooling law analysis, (**c**) result of peak logarithmic second derivative method, (**d**) result of frame subtraction method.

The three methods described in Section 3 were applied and the results are presented in Figure 9. Figure 9a shows a single frame of the raw thermal image, which was taken at the mid-point of the transient events (12:20:00) with the colour bar showing the range of the temperature. The single frame raw thermal image presented in Figure 9a shows high thermal contrast between the soil region and the air region, defects in the soil region are visible, but defects in the air region are not visible.

Figure 9b shows a map of the cooling constant obtained from Newton’s cooling law method. The boundary between the soil and air regions can again be clearly identified on the map. However, the defects in the air region are not visible, and there is only a faint indication of defects 4–6 in the soil region.

Figure 9c shows a map of the peak value of the logarithmic second derivative of the membrane temperature. This map shows a contrast between the soil region and the air region, but the subsurface defects could not be detected except for faint indications of defects 5 and 6 in the soil substrate.

The results from the frame subtraction method are shown in Figure 9d. This method was implemented with the first frame (corresponding to $t_{0}$ in Equation (11)) taken at the start of the transient event, the final frame at the end of the transient event at approximately 12:27:00, and with a time step of Δt=10 s. Most of the subsurface defects can be identified (specifically defects 2, 3, 5, 6 in the soil region and defects 8, 9, 11, 12 in the air region), and the colour contrast can be considered to provide an indication of the defect depth, in view of the observation that the strength of contrast increases with decreasing membrane thickness. However, the contrast between substrates is reduced due to the altered threshold that has been employed in this image display to highlight the defects.

These results demonstrate the advantages of analysing the entire thermal transient profile rather than a single image frame for detecting subsurface defects, even in the presence of different substrates. The merits and limitations of the three transient analysis methods are summarised in Table 8, which can be compared with Table 7 for Case 1. It appears that a combination of the three analysis methods can best achieve the objectives of identifying the different substrates, as well as detecting subsurface defects when in contact with different substrates. For example, maps of the cooling constant from Newton’s cooling law method and of the peak logarithmic second derivative can most clearly distinguish the substrates beneath the membrane, whereas the map from the frame subtraction method can most clearly detect subsurface defects when in contact with either the soil, water, or air region. However, a further systematic study of the process parameters is required to optimise the implementation of these techniques in practical applications.

## 5. Conclusions

This paper presents new concepts and algorithms for the quasi-active thermography inspection of an HDPE membrane that is in contact with various substrates. This quasi-active approach relies on naturally occurring variations in solar intensity due to cloud cover events to generate temperature transients suitable for thermographic analysis. A laboratory-scale experimental evaluation of this approach was undertaken. The experimental results verified that cloud shadings that result in reduced solar intensity can lead to identifiable temperature decay transients. It is determined that only transients resulting from solar intensity reductions of more than 400 W/m^2^ lasting for more than 1 min, should be employed for analysis. Three transient thermal image processing methods were investigated based on (i) a map of the cooling constant obtained by curve-fitting the temperature decay curve to an assumed exponential decay, in accordance with Newton’s law of cooling; (ii) a map of the peak value of the peak logarithmic second derivative of temperature; (iii) a frame subtraction method that employs the absolute value of the temperature differences between successive frames at a prescribed time interval. The results were compared with those from a single-frame thermal image, to assess their relative capability to identify various substrates and to defect subsurface defects on different substrates. The key findings of this study are summarised as:

- Compared to the map of temperature obtained from a single frame of the raw thermal image, the analysis based on the transient cooling process of membranes can better determine the presence of subsurface defects. The surface temperature of the membrane can be used to distinguish the substrate medium (soil, water, and air) and subsurface defects in the membrane;
- The three different thermal transient analysis methods have their own merits and limitations so that a combination of the three appears to be most suitable;
- The modified peak logarithmic second derivative and Newton’s cooling law methods show reasonable performance in distinguishing different substrates under the membrane cover, but the defect profiles are comparatively hard to identify on different substrates;
- The frame subtraction method with a predefined time interval provides the best indication of subsurface defects, especially in the presence of soil and air substrates, and the contrast values also correlate with the depth of the defects, thereby suggesting that a quantitative evaluation of defect severity may be possible.

While these results are very promising, further work including, in particular, detailed computational modelling, is still required to optimise the selection of parameters for the various approaches. A bigger area of thermal imaging monitoring from a greater distance is should also be conducted in future work.

## Figures and Tables

**Table 1:** Technical data of FLIR A615 IR camera.

Characteristic	Specification
Detector array size	640 × 480 pixels
Field of view (FOV)	25° × 19°
Minimum focus distance	0.25 m
Noise equivalent temperature difference (NETD)	<0.0 °C @ + 30 °C (+86 °F)/50 50 mK
Frame rate	3–50 Hz
Spectral range	7.5–14 μm
Accuracy	±2 °C
Operating temperature	−40–150 °C
Detector type	Focal plane array (uncooled microbolometer)

**Table 2:** Technical data of Apogee SP-110 pyranometer.

Characteristic	Specification
Sensitivity	0.2 mV/Wm^−2^
Calibrated output range	0–400 mV
Field of view	180°
Mass	90 g
Operating temperature	−40–70 °C
Response time	Less than 1 ms

**Table 3:** Technical data of Fluke 287 multimeter thermal probe.

Characteristic	Specification
Instrument model	Fluke 287 multimeter thermal probe
Operating temperature	−20–55 °C
Mass	871 g
temperature resolution	0.1 °C
Accuracy	±1%
Response time	Less than 1 ms

**Table 4:** Summary of material properties in the experiment.

Material	Density (kg/m^3^)	Thermal Conductivity (W/m*K)	Specific Heat (J/kg*K)	Thermal Diffusivity (mm^2^/s)
HDPE geomembrane	940 [33]	0.44 [33]	1900 [33]	0.246
Air (at 20 °C)	1.2754	0.138	1000	19
Water (at 20 °C)	997	0.6	4184	0.144
Soil	1350 [31]	0.47 [34]	1900 [35]	0.183
Scum	913 [32]	0.5 [27]	1400 [36]	0.391

**Table 5:** Dimensional details for defects shown in Figure 4a.

Defects Number	Defect Length (cm)	Defect Width (cm)	Defect Thickness (mm)	Location of Defects
1	5	0.5	0.5	Water region
2	5	0.5	1	
3	10	0.5	0.5	
4	10	0.5	1	
5	5	1	0.5	Air region
6	5	1	1	
7	10	1	0.5	
8	10	1	1	

**Table 6:** Dimensional details for defects shown in Figure 4b.

Defects Number	Defect Length (cm)	Defect Width (cm)	Defect Thickness (mm)	Location of Defects
1	10	1	0.5	Soil region
2	10	1	1	
3	10	1	1.5	
4	10	2	0.5	
5	10	2	1	
6	10	2	1.5	
7	10	1	0.5	Air region
8	10	1	1	
9	10	1	1.5	
10	10	2	0.5	
11	10	2	1	
12	10	2	1.5	

**Table 7:** Summary of merits and demerits of each method in Case 1.

	Air region and Water Region Identification	Defects Identification on Air Region	Defects Identification on Water Region	Image Quality
Raw thermal image	Yes	Barely visible	Barely visible	Clear
Newton’s cooling law method	Yes	Yes	Yes	Clear
LPSD method	Yes	Yes	Barely visible	Clear
Frame subtraction method	Yes	Yes	Barely visible	Clear

**Table 8:** Summary of merits and demerits of each method in Case 2.

	Air region and Water Region Identification	Defects Identification on Air Region	Defects Identification on Soil Region	Image Quality
Raw thermal image	Yes	No	4–6 are visible	Clear
Newton’s cooling law method	Yes	No	4–6 are visible	Clear
LPSD method	Yes	No	5–6 are visible	Clear
Frame subtraction method	Yes	Yes	Yes	Not clear

## Data Availability

The data presented in this study are available on reasonable request from the corresponding author.
